# The Avian Head Induces Cues for Sound Localization in Elevation

**DOI:** 10.1371/journal.pone.0112178

**Published:** 2014-11-12

**Authors:** Hans A. Schnyder, Dieter Vanderelst, Sophia Bartenstein, Uwe Firzlaff, Harald Luksch

**Affiliations:** 1 Chair of Zoology, Technische Universität München, Munich, Germany; 2 School of Biological Sciences, University of Bristol, Bristol, United Kingdom; 3 Active Perception Lab, University Antwerp, Antwerp, Belgium; University of Auckland, New Zealand

## Abstract

Accurate sound source localization in three-dimensional space is essential for an animal’s orientation and survival. While the horizontal position can be determined by interaural time and intensity differences, localization in elevation was thought to require external structures that modify sound before it reaches the tympanum. Here we show that in birds even without external structures like pinnae or feather ruffs, the simple shape of their head induces sound modifications that depend on the elevation of the source. Based on a model of localization errors, we show that these cues are sufficient to locate sounds in the vertical plane. These results suggest that the head of all birds induces acoustic cues for sound localization in the vertical plane, even in the absence of external ears.

## Introduction

In vertebrates, localization of sound in the horizontal plane is primarily achieved by comparing its phase and intensity differences between both ears (IPDs and IIDs, respectively) [1]. In contrast, sound localization in the vertical plane requires structures that induce spectral cues by modifying the sound before it reaches the tympanum [2]. In mammals this is typically achieved by external ears (pinnae) [3]. Due to their complex morphology, pinnae absorb, reflect and diffract sound depending on direction and frequency. Thus, the sound that reaches the tympanum has characteristic notches and peaks in the frequency spectrum, which are used for localization in elevation [1], [4]. In the barn owl, a nocturnal avian predator that strongly relies on auditory localization of prey [5], both the facial ruff and a vertical offset of the outer ear openings introduce intensity differences along the vertical plane [6]. This allows for localization in elevation [7], [8], especially in front of the animal [9]. Together with the frontally shifted position of the eyes, this is seen as a unique adaptation for hunting under dim light conditions. As virtually all other birds lack such specializations, they were considered largely incapable of sound localization in elevation [10]–[12], even though this was obviously at odds with the richly structured three-dimensional world of birds [13].

However, a possible role of the bird’s head in creating cues for sound localization in elevation was previously underestimated. The animal’s head will also absorb, reflect and diffract sound depending on sound direction and frequency. All of these modifications are described by the head related impulse response (HRIR) [14]. To prove the existence of direction-dependent peaks and notches in the sound spectrum even in the absence of pinnae, we investigated the HRIR in three bird species which lack external ears. To characterize a general effect irrespective of life style or phylogeny, we selected the chicken (*Gallus gallus*), the rook (*Corvus frugilegus*) and the duck (*Anas platyrhynchos*). None of these species is an auditory specialist, nor are they closely related or share similar ecological niches. We calculated the HRIR by cross-correlating white noise stimuli presented from various positions with the signals recorded in the ear canal close to the tympanum.

## Results and Discussion

**Figure 1:**
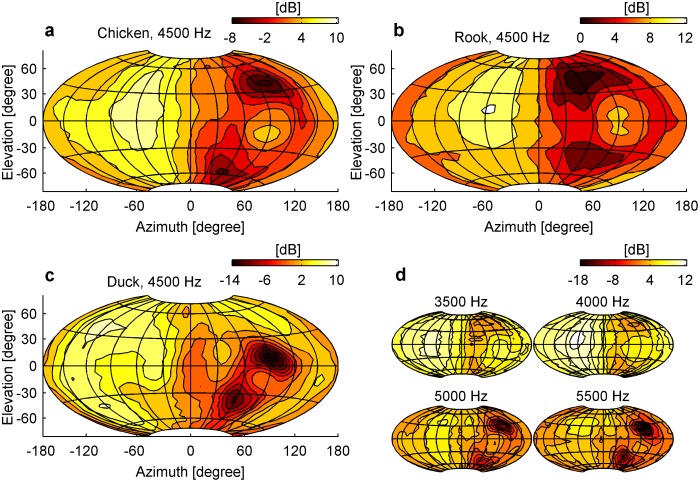
Bird heads [chicken (a, d), rook (b) and duck (c)] modify sound intensity dependent on elevation. Monaural gain [dB] is displayed at the right ear for multiple sound positions. Coordinates of 0° azimuth and 0° elevation face the beak, −90° azimuth and 0° elevation face the right ear. Sound intensity is projected according to the Hammer projection. Meshgrid spacing is 30°, iso-contourline spacing is 2 dB.

**Figure 2:**
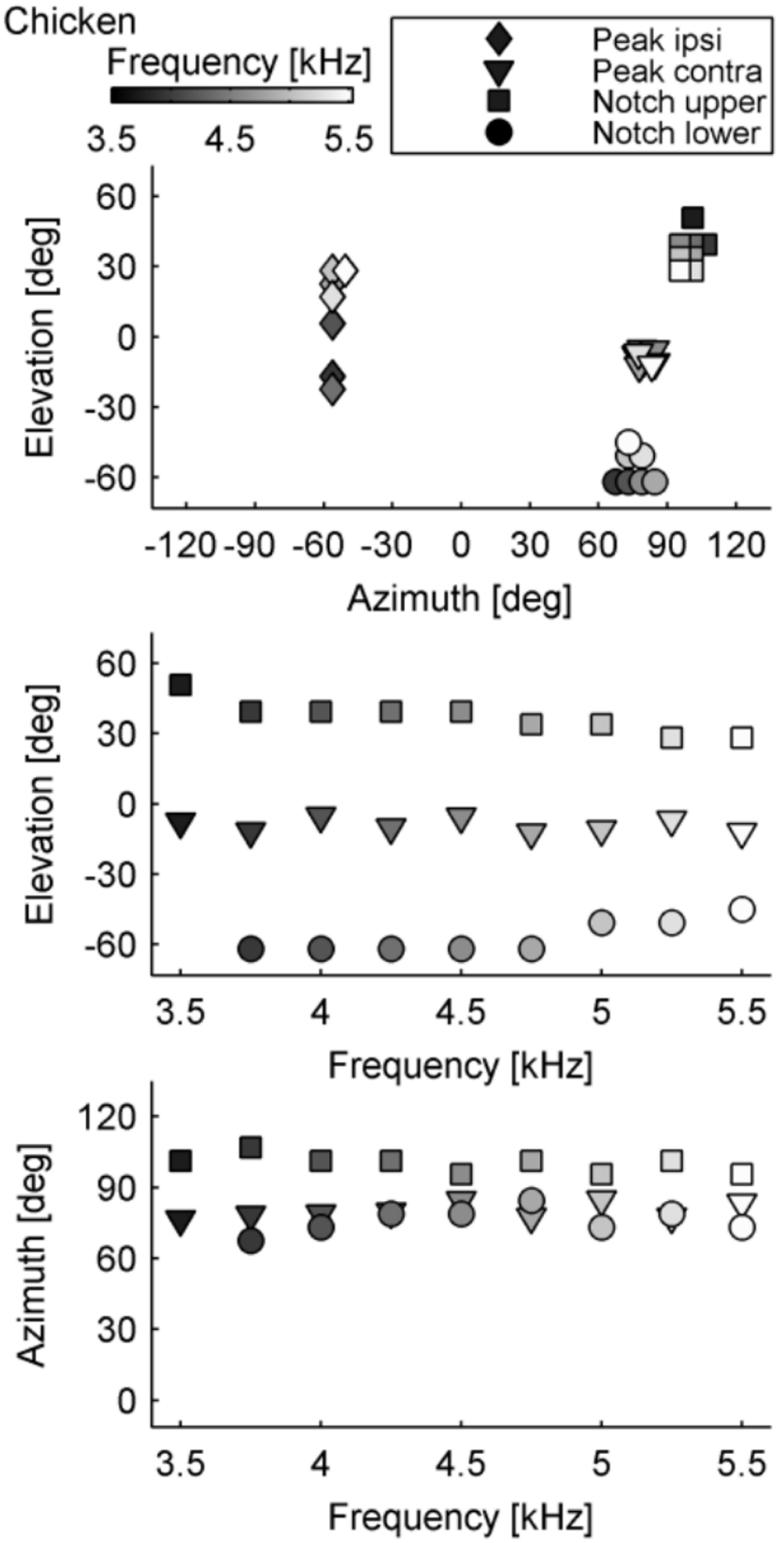
Contralateral peak position is stable over a wide frequency range. Positions of the minimum of the upper and lower notch and position of the maximum of the contralateral and ipsilateral peak from 3500 Hz to 5500 Hz in the chicken.

**Figure 3:**
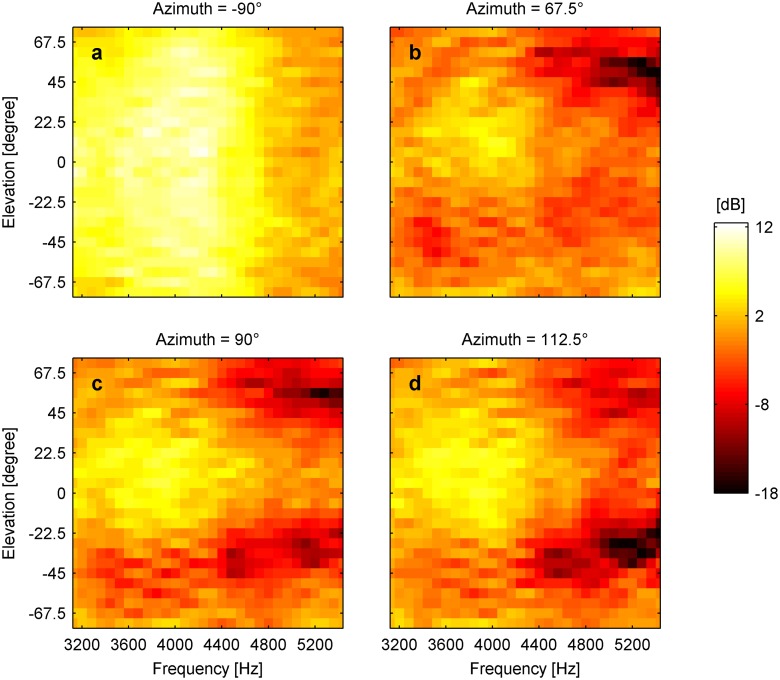
Monaural spectral cues change systematically along elevation. Monaural spectral cues between 3000 and 5500 Hz at a specified azimuth position for different elevation positions in the chicken.

Elevation-dependent sound modifications could be found in all avian heads (Figure 1a–c). As expected, sound presented ipsilateral to the examined ear resulted in a smooth intensity gain distribution along both the vertical and horizontal axis. Moving the sound source towards the contralateral side around the head resulted in decreasing sound levels, as the head increasingly shielded the ear from the sound source. However, when the sound was presented from the contralateral side, an intensity peak occurred at a distinct vertical and horizontal position. Along the vertical axis, intensity notches flanked the contralateral intensity peak in elevation (Figure 1d, Figure 2 and Figures S1–S2). This notch/peak/notch distribution was observed from 3500 Hz up to 5500 Hz (Figure 1d, Figures S3a–e, S4a–e and S5a–e), which is in the high frequency hearing range of the species examined [15], [16]. Therefore strong monaural spectral cues are present for contralateral azimuth positions and change systematically along elevation (Figure 3b–d, Figures S6b–d and Figure S7b–d). In contrast to this, for ipsilateral azimuth positions, the spectral profile did not change for different elevations and thus no spectral cues for sound localization would be available (Figure 3a, Figure S6a and Figure S7a). Such elevation dependent cues for contralateral sound were found in all three investigated species.

But what causes these cues? Interestingly, no complex mechanism or structure is needed to explain our observation. At a certain frequency range, spherical objects not only produce an acoustic shadow, but diffract sound to add up on the opposite side [17]. This well-known phenomenon (‘bright spot’) also applies to the head of birds, but has thus far never been linked to their hearing. The ‘bright spot’ in the midst of an acoustic shadow resembles the notch/peak/notch configuration we described above.

To quantify the contribution of the HRIR to sound localization, we used a model of vertebrate sound localization [18], [19]. Localization errors were estimated based on either the phase spectrum of the HRIR (pHRIR), the magnitude spectrum of the HRIR (mHRIR) or both combined (HRIR). Parameterizing the model with behavioural data in birds [20], we estimated the probability of sound originating from a given direction to be perceived as originating from any other direction. The localization error was expressed as the average angular deviation between the true origin of the sound and the perceived origin. Comparing the localization errors with and without mHRIR cues enables us to quantify how much the mHRIR contributes in reducing the ambiguity about the origin of a sound source. As mHRIRs were similar for the species tested (Figures S3 to S5), we selected the chicken data as representative.

**Figure 4:**
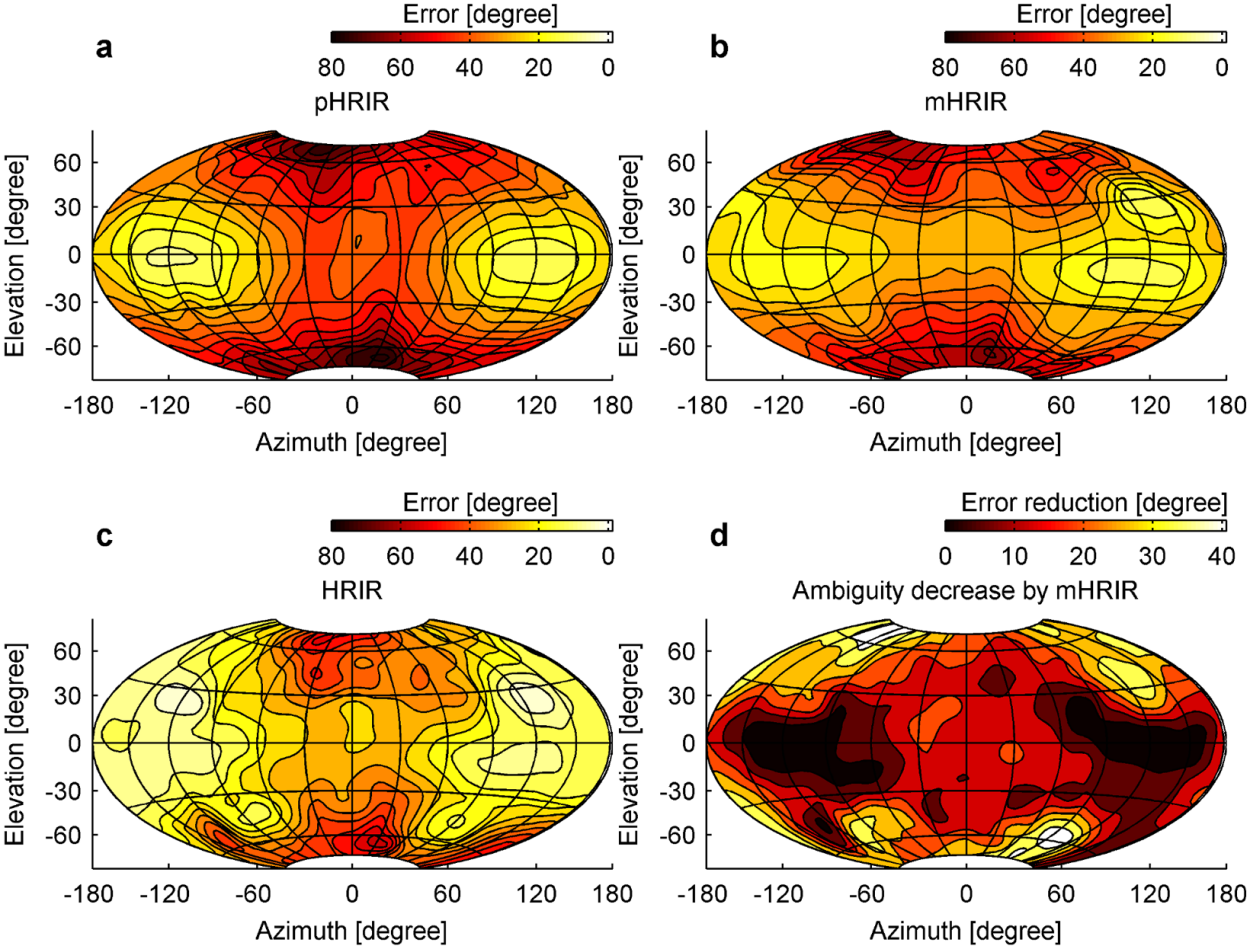
Localization error in elevation is lowest at spatial positions lateral to the head. The localization error in elevation is based on either the chicken‘s pHRIR (a), mHRIR (b) or both combined (c). The mHRIR‘s differential contribution to reduce elevation error (d) is calculated by subtracting (a) from (c). Spacing of error-contourline is 5°, map orientation in relation to the head and map projection are the same as in Figure 1.

As expected, pHRIR localization error in elevation is lowest at the sides of the head (Figure 4a). This is because the region where you find equal IPDs (called the ‘cone of confusion’, also indicated by the contourlines in Figure S8) decreases for lateral positions (Figure S8) [21]. The mHRIR reduces the localization error in elevation even further (Figure 4b). At the position where the notch/peak/notch distribution is located, the area of lowest error is enlarged. This effect is quantified in Figure 4c which shows the combined contribution of both mHRIR and pHRIR, and Figure 4d where the differential contribution of the mHRIR in reducing localization error is shown. Magnitude cues are most important for positions above 30° and below –30° in elevation for azimuth positions around ±90° (Figure 4d). From these positions the localization error based on phase is increasing again (Figure 4a) whereas the localization error based on the magnitude spectrum stays stable (Figure 4b). It is noteworthy that such an enlargement of low error regions resembles the effect of increased localization acuity in elevation generated by external structures like the feather ruff in barn owls [9]. The lowest predicted error across the complete space using the pHRIR, mHRIR or both was 9°, 7° and 7° respectively. However, the typical errors are higher than these values. For example, the average angular error in the frontal hemisphere (−90° to +90° azimuth) was 35°.

The increase in localization performance when using the mHRIR in addition to the pHRIR can be explained by the fact that (1) the mHRIR provides the chicken with two additional sources of information and (2) the assumption that the neural noise on the mHRIR and the pHRIR are uncorrelated.

The mHRIR provides the chicken with two sources of localization information in addition to the pHRIR. Indeed, the mHRIR can be rewritten as follows [19],
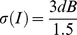



The first term in this denotes the IID. The second term is the average intensity (i.e. average power spectrum) across the two ears. Each of these two components of the mHRIR supplies the chicken with additional localization information.

First, a priori, in our data, 84% of the variation in the IID could be explained by the pHRIR. This indicates that the correlation between the pHRIR and mHRIR was large but not perfect. This means that errors are further reduced when adding the mHRIR. Second, for a sound source with a known spectrum, such as the white noise modelled in this paper or sound sources familiar to the bird, some localization information is encoded in the average spectrum across the two ears [18]. The correlation between the spectral component of the mHRIR and the pHRIR was only limited (26% of explained variance).

In addition to the two components of the mHRIR, there is a third reason why the mHRIR reduces the localization error. In our simulations, the noise on the neural encoding of the mHRIR and pHRIR cues is assumed to be uncorrelated. This is modelled by setting the off-diagonal elements of matrix 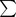 to zero. Therefore, even if both the IID and the average spectrum would correlate perfectly with the pHRIR (which is not the case) the mHRIR would increase performance because it is an additional channel with uncorrelated noise

**Figure 5:**
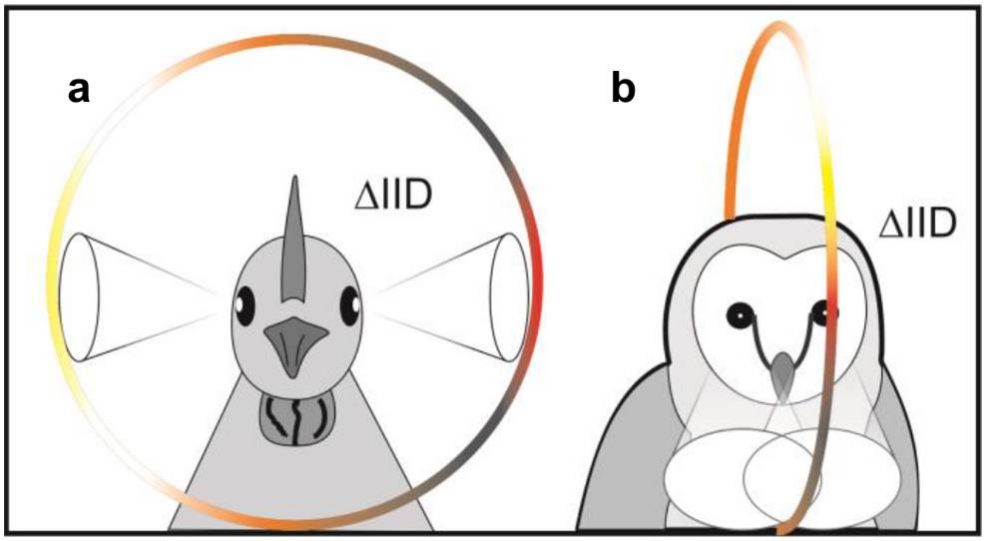
Schematic interpretation that directionality of vision and hearing align. Lateral eyed birds like *Gallus gallus* have access to elevation dependent IIDs on both sides (a) (Figures S4 to S6). Frontal eyed birds like *Tyto alba* however have access to their elevation dependent IIDs in front (b) [6]. Color indicates changing IID values.

Our data show that, even without external ear structures, birds have access to cues for sound localization in elevation. Simple physical diffraction around the bird’s head creates the spectral peaks and notches (Figure 1) which are necessary for sound localization in the vertical plane and are typically induced by external ears [4], [22]–[24]. These frequency-dependent intensity variations occur on the side contralateral to the sound source. Our model shows that a reliable sound localization in elevation is possible and in addition gives us for each sound position the respective localization accuracy. Lateral sounds are modified to such an extent that they are located with highest accuracy (Figure 4). Whereas a lateral focus for sound localization would misalign the acoustic and visual axis in frontal-eyed animals such as primates, for birds with laterally positioned eyes it is advantageous because it aligns the visual with the acoustic axis (Figure 5). This configuration directs both senses towards the same spatial location and thus facilitates object detection through multisensory integration [25]. Most avian species have lateral eye positions [26] that are essential to visually monitor the full extent of the environment – a crucial issue for animals that are preyed upon. For predators however, binocular vision and stereopsis are far more important [27]. Since the evolutionary pressure towards a larger binocular overlap conflicts with the lateral layout for auditory localization, it is conceivable that some predators (especially those that rely strongly on both vision and audition) developed alternative solutions. Through the formation of a feather ruff and asymmetrical ears, low-light predators such as the barn owl could again align both visual and auditory localization foci (Figure 5), maximizing hunting success at dusk and night.

Although evidence from behavioral experiments is still lacking, our results suggest that the majority of birds have retained the ancestral layout for sound localization in elevation – a solution that relies on a simple physical diffraction of sound by the head of the animal.

Even though we show that animals don’t need external ears to induce acoustic cues for sound localization in elevation, they still lack another feature that is distinct to pinnae. All these animals (birds, reptiles and some subterranean mammals) have relatively small heads and a low frequency hearing, which puts a limitation on the extent of their IIDs. Some comparable-sized mammals ‘solve’ this problem with large pinnae and high frequency hearing. This combination not only induces the spectral cues for sound localization in elevation, but also strong IIDs [3]. In contrast all animals which lack pinnae share an interaural canal that connects both middle ear cavities with each other. These internally coupled ears are thought to boost IIDs and IPDs internally. This could present a further solution to the challenge of small heads without pinnae [12].

## Methods

### Head related impulse response measurement

We conducted all experiments in a sound reduced environment. We measured the directionality of sound pressure transformation (HRIR, head related impulse response) in heads of three species of three different avian orders, Galliformes, Anseriformes and Passeriformes (*Gallus gallus*, *Anas platyrhynchos* and *Corvus frugilegus*, respectively). All tested specimen were full grown adults. We measured head widths of 30 mm for the chicken, 34 mm for the duck and 30 mm for the rook. The necks were still attached and intact, therefore any influence on the HRIR was included as well. The work was done in accordance with the *Directive 2010/63/EU of the European Parliament and of the Council of 22 September 2010 on the protection of animals used for scientific purposes*, but was not subject to official approval by the local authority the Regierung von Oberbayern. Chicken heads were obtained from the Versuchsstation Thalhausen, duck heads from the Geflügelhof Lugeder and the crow from the Klinik für Vögel, Reptilien, Amphibien und Zierfische of the Ludwig-Maximilians-Universität München. Chicken and duck heads were slaughterhouse waste. The crow was brought to the clinic as a casualty in an accident in Landkreis München. No animal was killed for the purpose of this study. Specimens were freshly dead or deep frozen. We fixated the heads in 4% paraformaldehyde solution for one week and made a small incision from behind towards the ear canal on both sides of the head. Then we inserted a small microphone (Knowles, EM-D65) through this incision into the hearing canal just in front of the tympanic membrane. To ensure that the microphone only received sound from the ear entrance it was placed into a tightly fitting metal tube. This tube was cemented into the incision and all remaining openings were sealed. In our experiments we did not investigate the influences of fixation on the HRIR. However in experiments with owls the fixation did not influence the HRIR measurements compared to anesthetized animals for frequencies below 7 kHz [28].

We placed the thus prepared head into the centre of a rotatable semicircular loudspeaker array. It has a diameter of 102 cm and is equipped with 27 speakers covering −73.125° to 73.125° in elevation in 5.625° steps. The array was rotated from –180° to 180° in 5.625° steps azimuth. We measured the directionality of hearing at a total of 1755 positions. The midpoint between both ears was in the centre of the semicircle. The beak faced its central speaker in its zero position, which is defined as elevation and azimuth 0°. Vertical positions of beak, ear entrances and the central speaker define the horizontal plane. This position resembles the head position observed under natural conditions.

We based the measurement of HRIRs on methods described in [14], [29]. We digitally generated white noise between 60–10000 Hz of 2 seconds duration (MATLAB R2013b, Florida, USA) and broadcasted it after DA conversion (Fireface 400, RME, samplerate 44100 Hz) sequentially from each of the 1755 possible speaker positions. Signal to noise ratio was about 50 dB. We then recorded the noise signal by the microphones inside both ear canals and cross-correlated it with the original signal to establish the impulse response. To cancel the influence of individual speakers and the tube/microphone assembly, we calibrated each speaker before every recording session with the tube/microphone assembly in the same positions as when implanted in the birds’ head under test conditions.

We cut the impulse responses from all positions with a rectangular window to remove any undesired reflection which might have originated from metal parts of the speaker array. We padded the impulse responses with zeroes to the final length of 256 points and performed a Fourier transformation (MATLAB). By dividing the Fourier transformation of the ear canal response by the Fourier transformation of the calibration response we obtained a proper HRIR. The FFT window of 256 points yielded a spectral line resolution of 172.3 Hz.

We processed all data with in-house developed programs written in MATLAB. We organized the mHRIR data, which is the magnitude spectrum of the HRIR, in a three-dimensional azimuth-by-elevation-frequency array. Data were smoothed in azimuth and elevation with a standard MATLAB function (Box convolution kernel (size = 3)). We graphed the data as two-dimensional contour plots (2 dB contour spacing) using a Hammer projection. We defined the sound intensity (dB) as the gain relative to the calibration measurement. By subtracting the mHRIR obtained from the right and the left ear we calculated interaural intensity differences (IIDs).

### Estimation of the localization error

We estimate the likelihood that a broadband noise burst (with a known spectrum) will be correctly localized with an approach that has been used to estimate the echolocation performance in bats [18], [30] and sound localization in humans [19]. In parallel, we estimate the errors birds will make in localizing white noise bursts. Noise bursts are commonly used in behavioural experiments [11]. This makes it interesting to model the localization of these particular stimuli although more complex stimuli could add additional cues and cue variability that might influence the localization performance of the model.

Our estimation of localization errors is based on the head related impulse response (HRIR) and the known temporal resolution and intensity discrimination of the common birds hearing apparatus. We estimate the localization errors in elevation. Moreover, localization performance is estimated including and not including the mHRIR. This allows us to assess the contribution of the mHRIR, which includes IIDs, in reducing the errors in the vertical plane. The parameters used in calculating the localization errors are derived from behavioural experiments [20]. In the following we describe the model used to estimate the localization errors.

Let 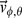 be the vector describing the HRIR as measured at the left and the right tympanic membrane for azimuth and elevation 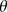. The parameter 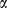 denotes the gain of the noise burst. The vector 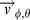 is constructed as given in the following equation,
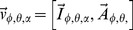



with
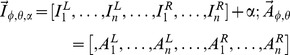



In this equation 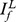 and 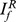 denote the sound intensity (in 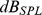) for frequency 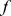 at the left and the right tympanic membrane respectively. Likewise, 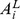 and 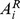 denote the phase (in radians) for frequency 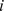 at the left and the right tympanic membrane respectively. These intensities have been normalized such that the maximum across all frequencies, positions and both ears 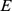 is zero (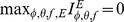). Values of 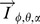 smaller than zero are set to zero as these are below the hearing threshold.

Due to noise, the hearing apparatus of the bird has a limited ability to encode the phases and intensities. Therefore, assuming Gaussian noise modelled by the covariance matrix 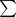, the hearing system will only have access to a noisy version of vector 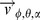 given by 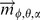 defined as follows,
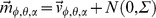



The probability of a vector 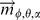 to originate from azimuth 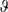 and elevation 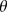 is given by,
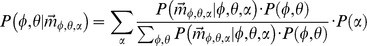



with
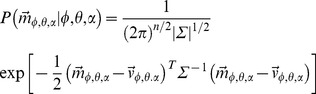



As the gain 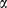 of the noise burst is not directly accessible to the animal, it is considered as a nuisance parameter that is removed from the equations by integration

The probability to perceive a noise burst originating from direction 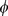, 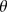 as coming from direction 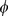
’, 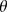
’ is given by 
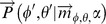
, I.e. 
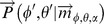
 averaged across many realizations of 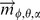. However, to reduce the computational load, we approximate 
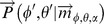
, as follows [see [18] for a justification],
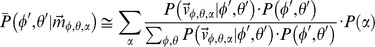



with
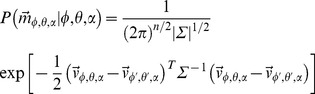



This equation allows us to estimate both the probability of correct localization and the expected localization errors. The probability of correct localization is given by 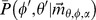 for 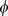 = 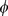
’ and 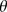 = 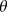
’. The localization errors in azimuth and elevation are given by,
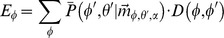



and
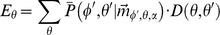



With 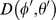 and 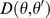 the great circle distance between the real azimuth 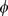 and elevation 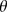 and each of the other azimuth 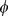
’ and elevation 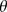
’ positions considered.

The calculation of the localization performance as outlined above depends on the HRIR and the covariance matrix modelling the additive noise. The HRIR for frequencies from 1000 Hz to 5500 Hz were used as they are consistent with the typical hearing range in birds [15]. We model the auditory channels of the bird using equivalent rectangular band pass filters (ERB) with a bandwidth of 500 Hz, which is a good fit for higher frequencies but is overestimating the bandwidth for lower frequencies [31]. As such, our model is more likely to underestimate the localization performance than to overestimate it. The spacing of the frequencies supports the assumption that the noise in different frequency channels is independent. To the best of our knowledge, there is no data suggesting the noise on the two neural channels is correlated. Correlated noise would imply that any overestimation (/underestimation) of the intensity at the left and right ear would be systematically associated with an overestimation (/underestimation) of the interaural phase difference. However, both neural channels are encoded by different neural substrates. While the magnitude of the signal at the left and the right ear are encoded on the level of the cochlear nucleus, the phase information (i.e. difference in phase at both ears) is encoded at the level of the nucleus laminaris using delay lines [32]. Moreover, IPD and intensity cues are processed independently in birds: intensity cues are found to not influence the encoding of the IPD [33]. This makes it unlikely that the noise of the two channels is correlated. Therefore, in the absence of any evidence suggesting differently, we model the noise independently. Therefore, the off-diagonal elements of 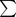 were set to 0. The diagonal elements of the matrix 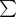 represent the additive noise on the intensities and phases. These values were deduced from behavioral experiments [20].

Welch and Dent (2011) measured interaural level and time difference discrimination thresholds in budgerigars. Interestingly, they report the discrimination thresholds for d’ = 1.5. The d'gives the distance between distributions in units of the standard deviation of the noise distribution. Therefore, these data allow to directly parameterize the matrix 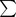. The minimal interaural time difference discrimination thresholds reported by Welch & Dent 2011 is about 20 µs at 2000 Hz, which implies Gaussian noise on the phase for frequency 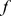 with a standard deviation given by, 
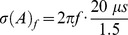
. Welch and Dent 2011 report a minimal discrimination threshold of 3dB. Therefore, the standard deviation of the noise on the intensity was set to 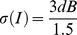. Finally, as the avian auditory system is unable to phase lock for frequencies above 4000 Hz [11] no phase information was included for frequencies above 4000 Hz.

## Supporting Information

Figure S1 **Contralateral peak position is stable over a wide frequency range.** Positions of the minimum of the upper and lower notch and position of the maximum of the contralateral and ipsilateral peak from 3500 Hz to 5500 Hz in the duck.
(TIF)

Figure S2 **Contralateral peak position is stable over a wide frequency range.** Positions of the minimum of the upper and lower notch and position of the maximum of the contralateral and ipsilateral peak from 3500 Hz to 5500 Hz in the rook.
(TIF)

Figure S3 (**a–e**) Monaural gain at the right ear and (**f–j**) interaural intensity differences (IIDs) of a chicken between 3500 and 5500 Hz.
(TIF)

Figure S4 (**a–e**) Monaural gain at the right ear and (**f–j**) interaural intensity differences (IIDs) of a duck between 3500 and 5500 Hz.
(TIF)

Figure S5 (**a–e**) Monaural gain at the right ear and (**f–j**) interaural intensity differences (IIDs) of a rook between 3500 and 5500 Hz.
(TIF)

Figure S6 **Monaural spectral cues between 3000 and 5500 Hz at a specified azimuth position for different elevation positions in the duck.**
(TIF)

Figure S7 **Monaural spectral cues between 3000 and 5500 Hz at a specified azimuth position for different elevation positions in the rook.**
(TIF)

Figure S8 **Interaural phase differences (IPDs) at 500, 1000, 2000 and 4000 Hz in the chicken.** Spacing of iso-contourline is 10°, map orientation in relation to the head and map projection are the same as in Figure 1.
(TIF)

Chicken S1 **Head related impulse response (HRIR) and head related transfer function (HRTF) for the left and right ear of a female adult chicken.** The sampling rate (fs) is 44100 Hz, fast Fourier transform points (NFFT1) are 512. The azimuth (allaz) ranges from −180° to 180°, in 65 steps with a spacing of 5.625°. The elevation (allele) ranges from −73.125° to 73.125°, in 27 steps with a spacing of 5.625°.
(MAT)

Duck S1 **Head related impulse response (HRIR) and head related transfer function (HRTF) for the left and right ear of an adult duck.** Sampling rate, fast Fourier transform points, azimuth and elevation range, steps and spacing are the same as in Chicken S1.
(MAT)

Rook S1 **Head related impulse response (HRIR) and head related transfer function (HRTF) for the left and right ear of an adult rook.** Sampling rate, fast Fourier transform points, azimuth and elevation range, steps and spacing are the same as in Chicken S1.
(MAT)
