# Correction: The Avian Head Induces Cues for Sound Localization in Elevation

**DOI:** 10.1371/journal.pone.0118325

**Published:** 2015-02-26

**Authors:** The PLOS ONE Staff

In the Results and Discussion section, there is an error in the equation for mHRIR following the sixth paragraph. Please view the complete, correct equation here:

$${\vec{I}}_{\varphi ,\theta ,\alpha }=\left[{\vec{I}}_{\varphi ,\theta ,\alpha }^{R}-{\vec{I}}_{\varphi ,\theta ,\alpha }^{L}\right]+\frac{\left[{\vec{I}}_{\varphi ,\theta ,\alpha }^{L}+{\vec{I}}_{\varphi ,\theta ,\alpha }^{R}\right]}{2}$$
