# Supplementary figures and images for: The Avian Head Induces Cues for Sound Localization in Elevation
Source: PLoS One. 2014 Nov 12;9(11):e112178. doi: 10.1371/journal.pone.0112178 (PMC4229125; doi:10.1371/journal.pone.0112178)

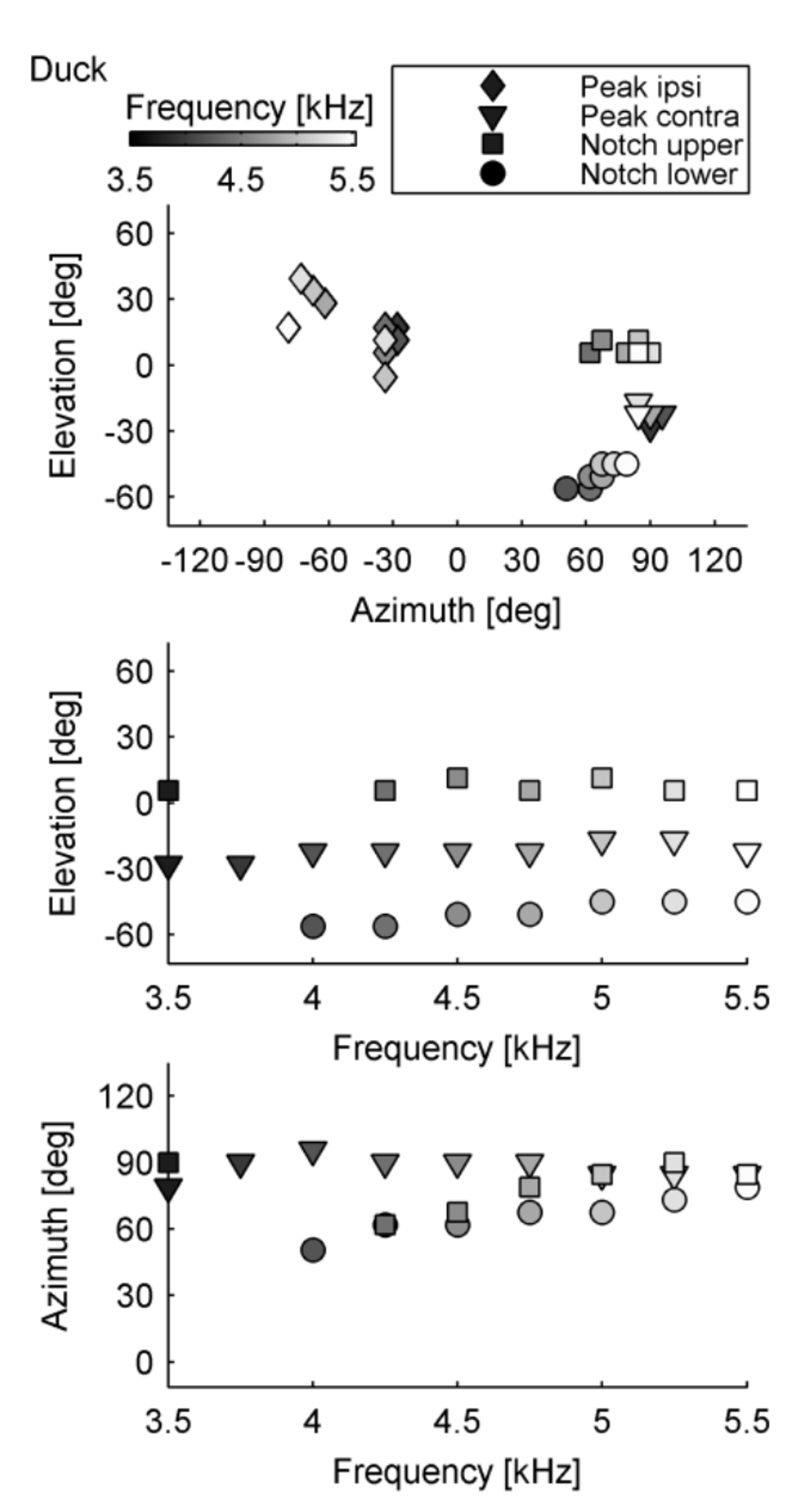

Supplement: Figure S1 — Contralateral peak position is stable over a wide frequency range. Positions of the minimum of the upper and lower notch and position of the maximum of the contralateral and ipsilateral peak from 3500 Hz to 5500 Hz in the duck. (TIF) [file pone.0112178.s001.tif]

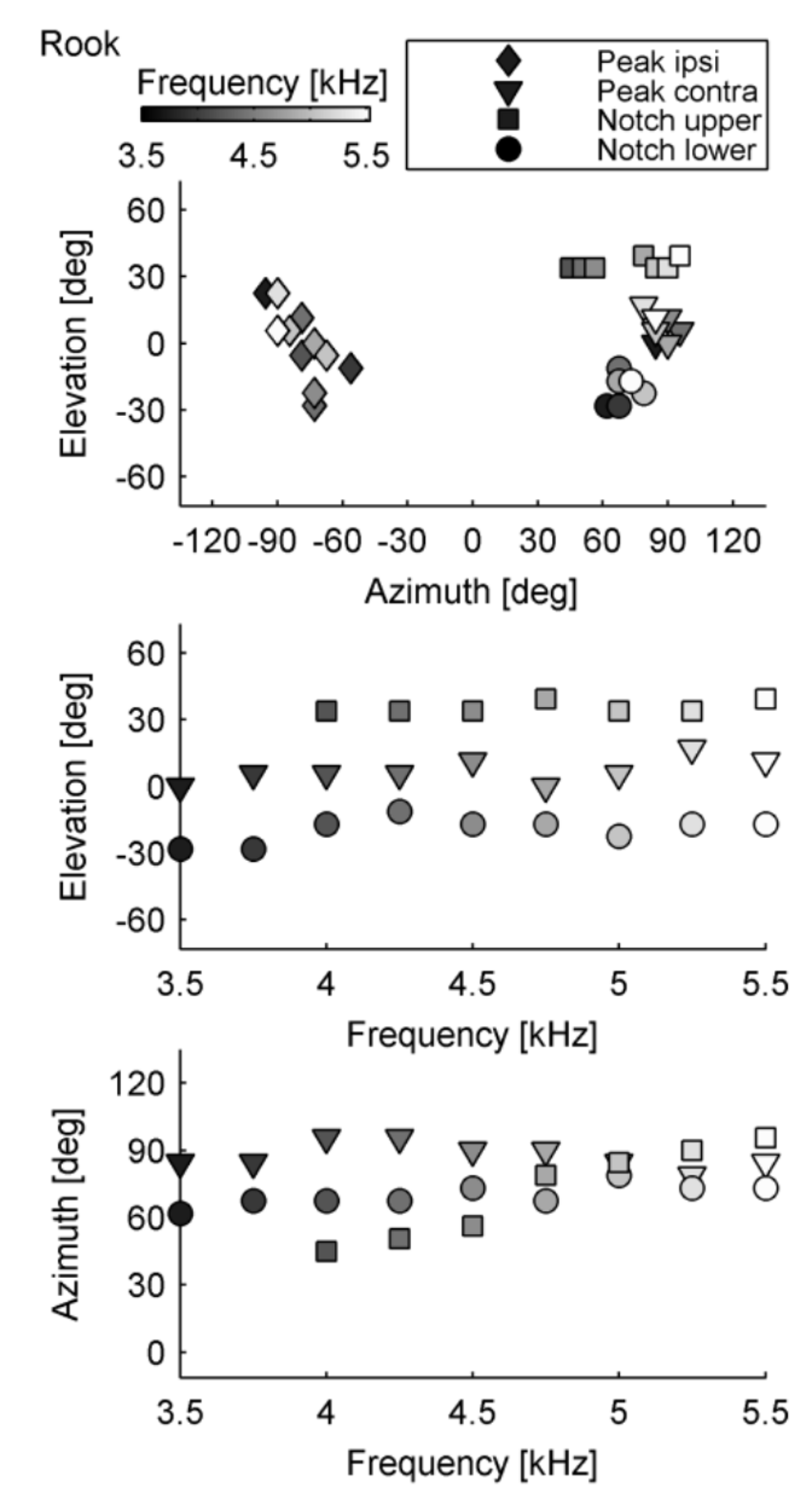

Supplement: Figure S2 — Contralateral peak position is stable over a wide frequency range. Positions of the minimum of the upper and lower notch and position of the maximum of the contralateral and ipsilateral peak from 3500 Hz to 5500 Hz in the rook. (TIF) [file pone.0112178.s002.tif]

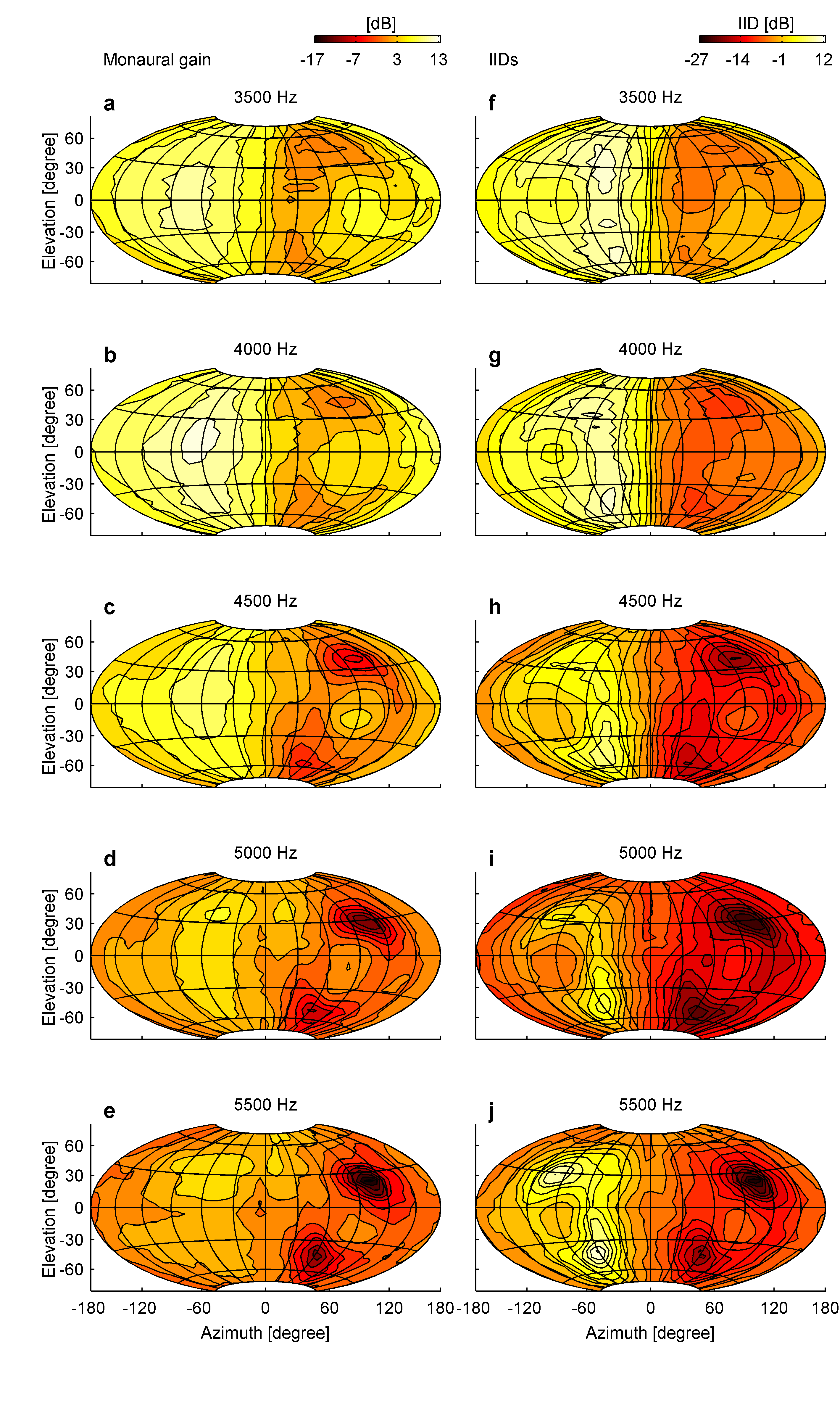

Supplement: Figure S3 — (a–e) Monaural gain at the right ear and (f–j) interaural intensity differences (IIDs) of a chicken between 3500 and 5500 Hz. (TIF) [file pone.0112178.s003.tif]

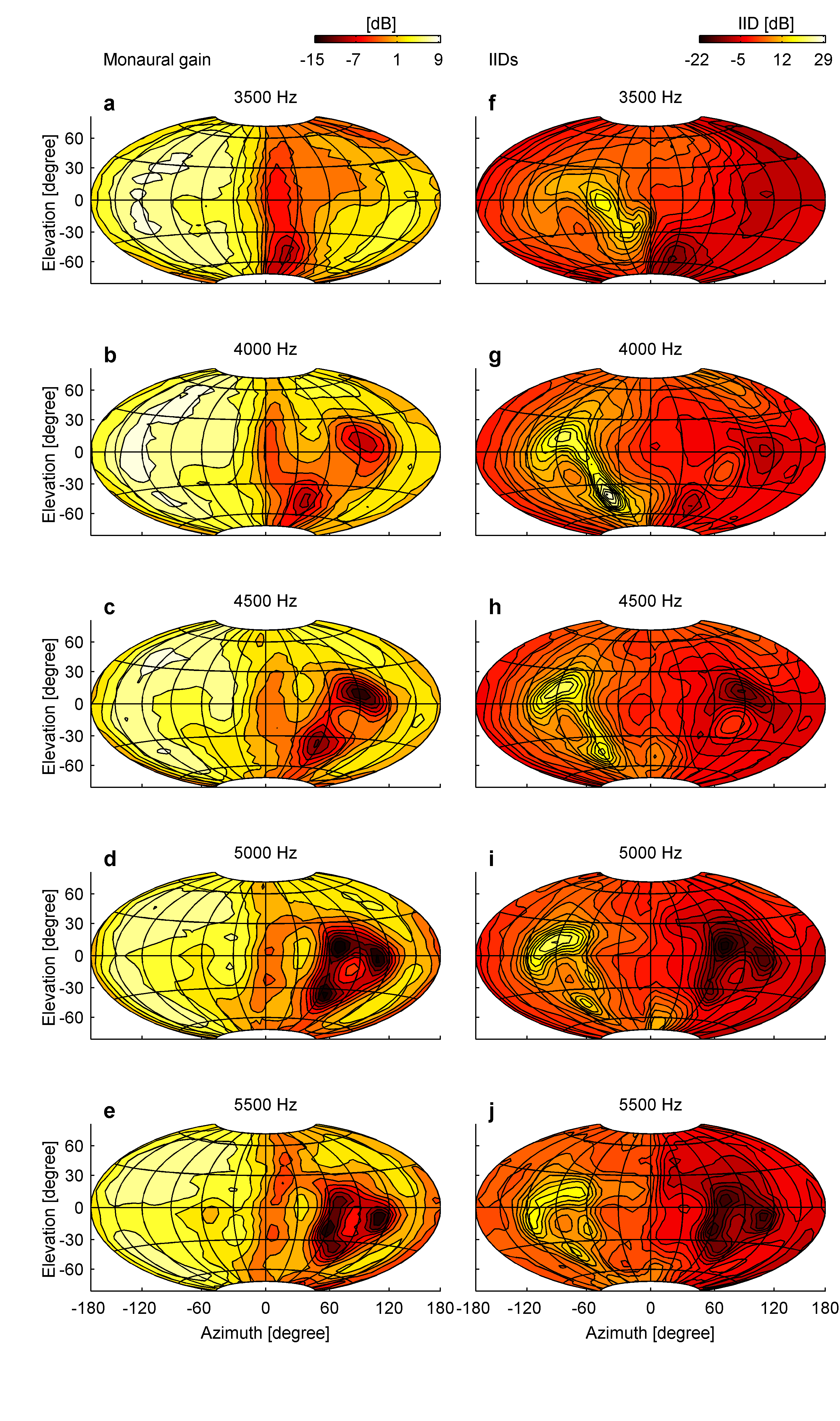

Supplement: Figure S4 — (a–e) Monaural gain at the right ear and (f–j) interaural intensity differences (IIDs) of a duck between 3500 and 5500 Hz. (TIF) [file pone.0112178.s004.tif]

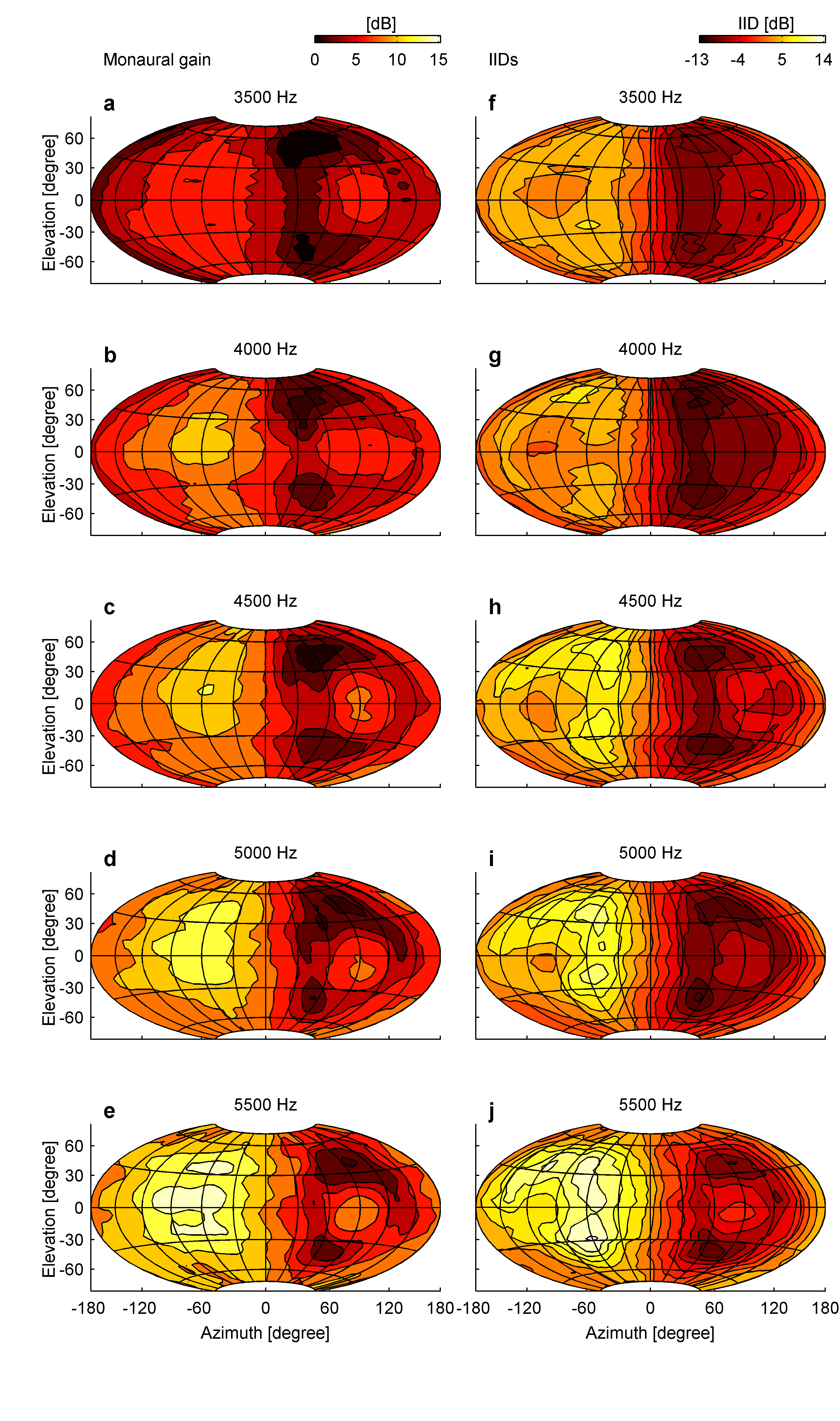

Supplement: Figure S5 — (a–e) Monaural gain at the right ear and (f–j) interaural intensity differences (IIDs) of a rook between 3500 and 5500 Hz. (TIF) [file pone.0112178.s005.tif]

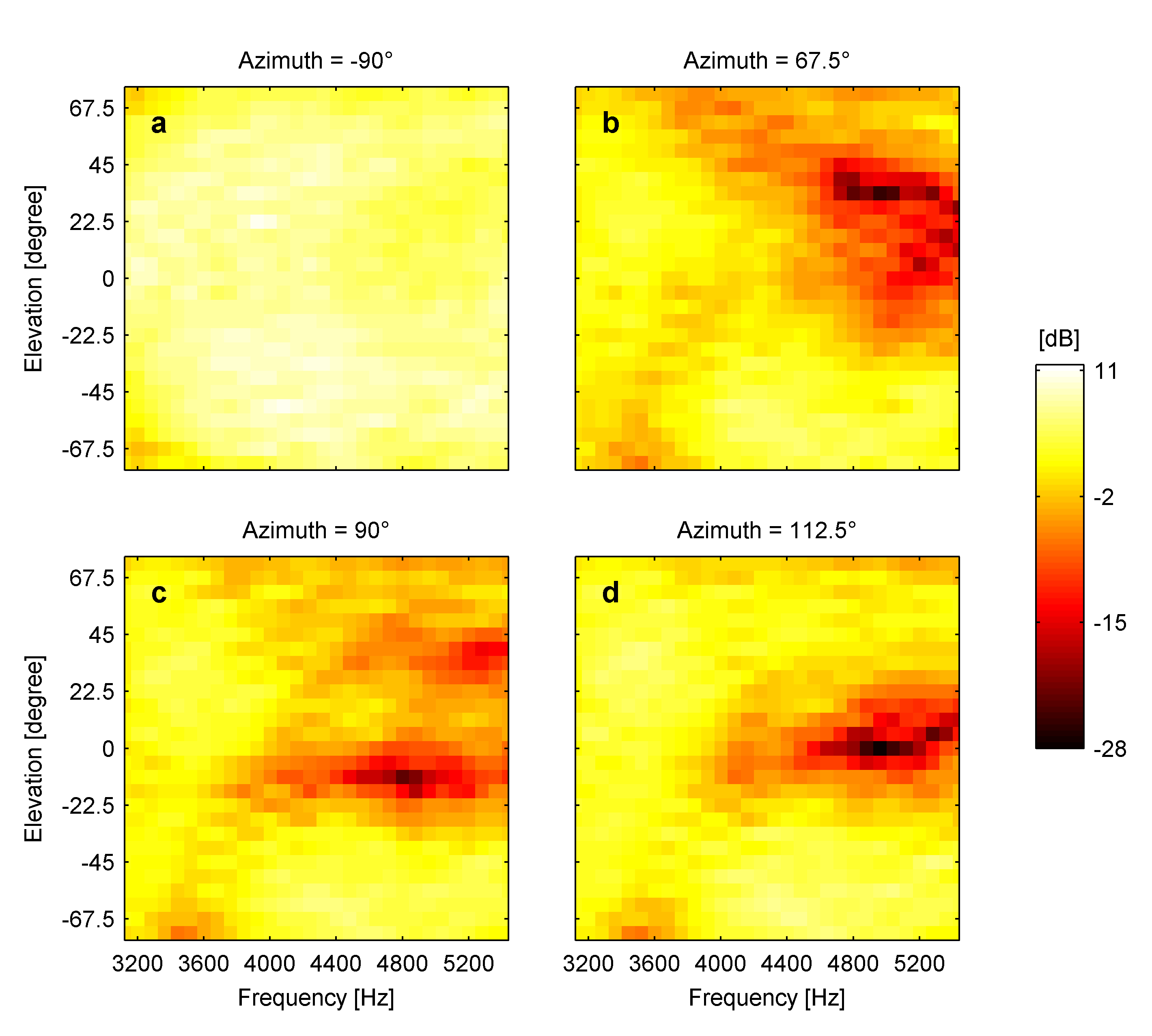

Supplement: Figure S6 — Monaural spectral cues between 3000 and 5500 Hz at a specified azimuth position for different elevation positions in the duck. (TIF) [file pone.0112178.s006.tif]

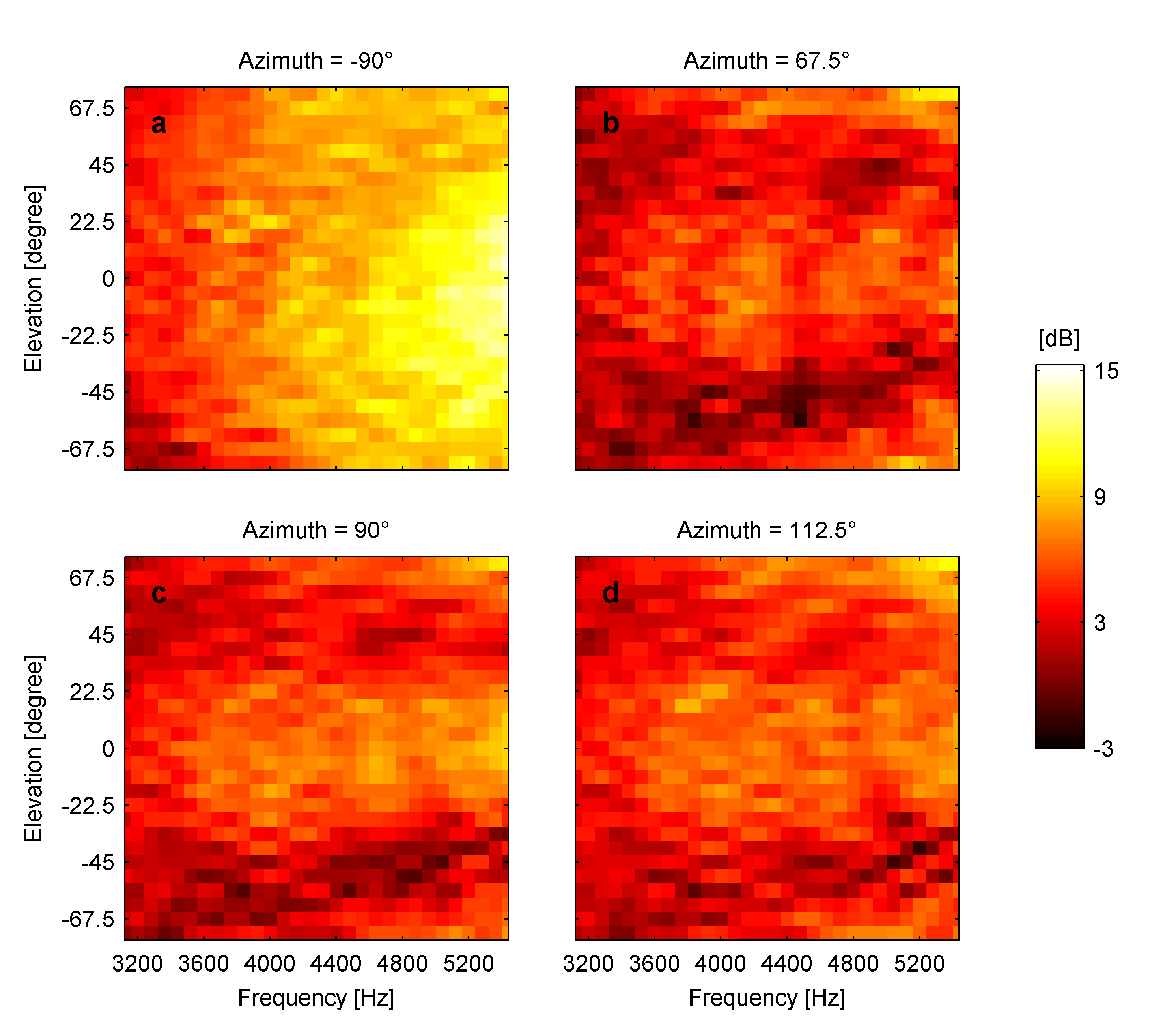

Supplement: Figure S7 — Monaural spectral cues between 3000 and 5500 Hz at a specified azimuth position for different elevation positions in the rook. (TIF) [file pone.0112178.s007.tif]

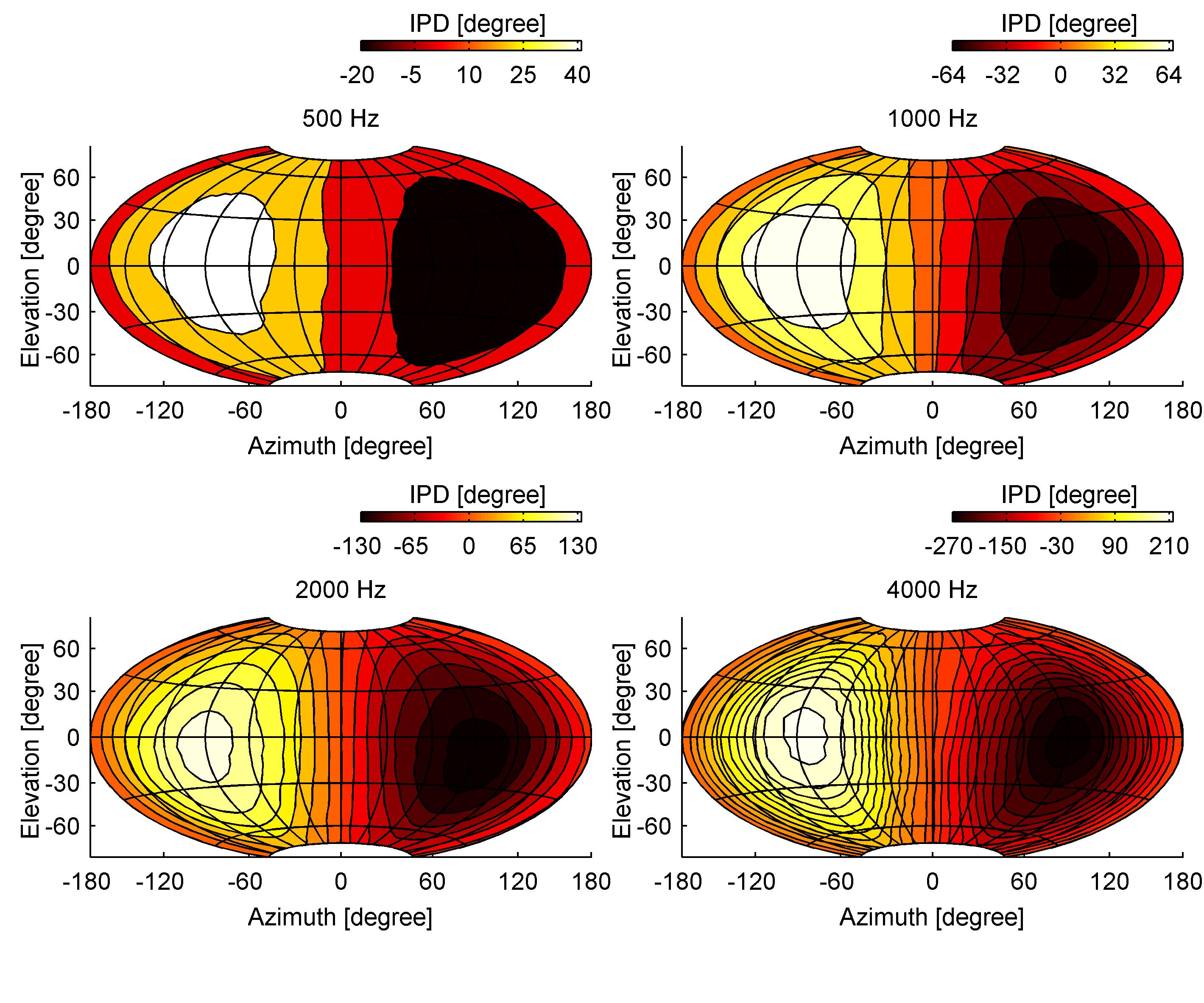

Supplement: Figure S8 — Interaural phase differences (IPDs) at 500, 1000, 2000 and 4000 Hz in the chicken. Spacing of iso-contourline is 10°, map orientation in relation to the head and map projection are the same as in Figure 1. (TIF) [file pone.0112178.s008.tif]
